# The role of female hormones on lung function in chronic lung diseases

**DOI:** 10.1186/1472-6874-11-24

**Published:** 2011-06-03

**Authors:** Anthony Tam, Don Morrish, Samuel Wadsworth, Delbert Dorscheid, SF Paul Man, Don D Sin

**Affiliations:** 1 The UBC James Hogg Research Centre, Providence Heart + Lung Centre & Department of Medicine, University of British Columbia (UBC), Vancouver, BC, Canada; 2 Department of Medicine, The University of Alberta, Edmonton, AB, Canada

**Keywords:** lung function, menstrual cycle, sex hormones, asthma, cystic fibrosis, COPD

## Abstract

**Background:**

The prevalence, morbidity, and mortality of inflammatory lung diseases such as asthma, chronic obstructive pulmonary disease (COPD) and cystic fibrosis (CF) are increasing in women. There is a dearth of data on the biological mechanisms to explain such observations. However, some large epidemiologic studies suggest that lung function fluctuates during the menstrual cycle in female patients with airways disease but not in women without disease, suggesting that circulating estradiol and progesterone may be involved in this process.

**Discussion:**

In asthma, estradiol shuttles adaptive immunity towards the T_H_2 phenotype while in smokers estrogens may be involved in the generation of toxic intermediate metabolites in the airways of female smokers, which may be relevant in COPD pathogenesis. In CF, estradiol has been demonstrated to up-regulate MUC5B gene in human airway epithelial cells and inhibit chloride secretion in the airways. Progesterone may augment airway inflammation.

**Summary:**

Taken together, clinical and in-vivo data have demonstrated a sex-related difference in that females may be more susceptible to the pathogenesis of lung diseases. In this paper, we review the effect of female sex hormones in the context of these inflammatory airway diseases.

## Background

### The sex differences in the epidemiology of asthma, COPD and CF

**Figure 1:**
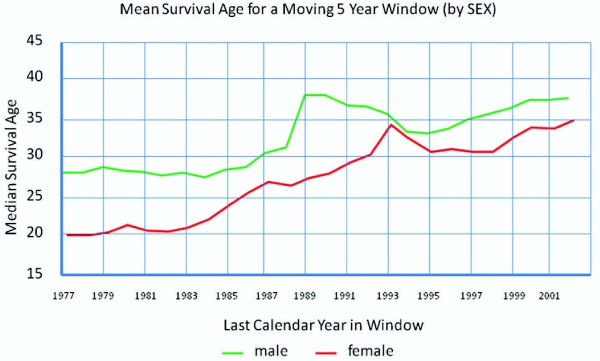
**Median survival ages in both sexes in CF from 1977-2002**. Obtained from the Canadian Cystic Fibrosis Foundation, Report of the Canadian Patient Data Registry 2002, Toronto, Ontario [8].

There is an epidemic of inflammatory lung diseases such as asthma, chronic obstructive pulmonary disease (COPD) and cystic fibrosis (CF). Although these conditions have distinct pathophysiologies, women for largely unknown reasons are increasingly becoming more prevalent and experiencing excess morbidity and mortality from these disorders. For instance, in the United States (U.S.) and elsewhere, more than 60% of all adult patients with asthma are women, and female compared to male asthmatics are 50% more likely to have physician visits, 35% more likely to experience hospitalizations and 40% more likely to die from asthma [1]. Although men have higher prevalence of COPD than women, the increased rates of cigarette smoking in females within the last several decades have been associated with steadily increasing rates of COPD in females [2]. In 2000, for the first time, the number of women dying of COPD in the United States surpassed the number of men [3]. Even in cases in which cigarette smoking is implicated, women develop COPD after smoking fewer number of cigarettes per lifetime (i.e. less pack-years of smoking) [4] and are two to three times more likely to experience hospitalization than are male patients [5]. Among first-degree relatives of patients with severe COPD, female smoking relatives demonstrate significantly lower lung function compared to male smoking relatives [6]. In patients with severe COPD with oxygen dependence, women have a 50% increase in the risk of mortality compared to men [7]. Finally, although CF is a rare genetic disorder that affects both men and women, for largely unclear reasons, female patients have shorter life expectancy compared to male patients (Figure 1). Collectively, these epidemiologic data suggest that female gender is a significant risk factor for morbidity and mortality in inflammatory lung diseases and raise the possibility that sex-related hormones may be important in mediating disease progression in asthma, COPD and CF, though the mechanisms by which this occurs may differ between these disorders. In this paper, we will examine the potential role of estrogen and progesterone in the pathogenesis of these disorders.

### Biology of sex hormones

#### Estrogen and progesterone

**Figure 2:**
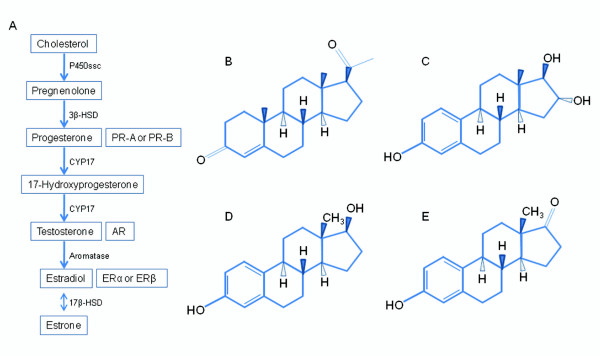
**Used with permission from the American Physiological Society**. A) Overview of the sex steroid hormone biosynthetic pathway and associated nuclear receptors. P450ssc, P450-linked side chain cleaving enzyme; CYP17, cytochrome P450 17; 3β-HSD, 3β-hydroxysteroid dehydrogenase; 17β-HSD, 17β-hydroxysteroid dehydrogenase; PR, progesterone receptor; AR, androgen receptor; ER, estrogen receptor. B) Progesterone, C) estriol, D) estradiol, E) estrone [9].

Steroid hormones are primarily synthesized in the gonads, adrenal glands, and the feto-placental unit [9]. Cholesterol is the common precursor of all steroid hormones where it is first converted to pregnenolone by P450-linked side chain cleaving enzyme (P450ssc) (Figure 2A). Pregnenolone is then converted to progesterone, which is used to synthesize androgens and estrogens [10]. Estrogens are derived from androgens by the addition of an aromatic A ring, through a reaction that is catalyzed by the enzyme aromatase [10,11]. Estrogen and progesterone are multi-ringed structures with distinct functional groups (Figure 2B, C, D, E). Unlike progesterone, there are three major naturally occurring estrogens in women: 1) estriol, 2) estradiol, and 3) estrone. In humans, estriol is the predominant estrogen in pregnant women, while estradiol is the predominant form in the non-pregnant premenopausal women and estrone is the predominant estrogen in the menopausal females. Sex steroid hormones act via their own unique receptors: estrogen receptor (ER-α or ER-β), progesterone receptor (PR-A or PR-B), and an androgen receptor (AR) [12]. Estradiol binds with a higher affinity to ER than its metabolic products such as estrone and estriol [13]. All sex steroid hormone receptors have been shown to be expressed in lung tissue [14,15].

#### Sex hormones and menstrual cycle

Four main hormones characterize the menstrual cycle: estradiol (E2), progesterone (P), luteinizing hormone, and follicle stimulating hormone. On average, the menstrual cycle is 28 days and is divided in two phases: the follicular (Day 1-13) and the luteal phase (Day 14-28). The onset of ovulation is defined by a surge in estradiol on day 14. Menstruation and the late luteal phase are characterized by low serum levels of estradiol (~0.15 nM) and progesterone (~9.54-31.81 nM); whereas ovulation is marked by high circulating levels of estradiol (~0.37-1.47 nM) and low levels of progesterone (~0.95-9.54 nM) (Table 1). During the luteal phase, estradiol levels range from 0.15-0.92 nM, whereas progesterone levels increase from 9.54-31.81 nM and drop back to lower levels prior to menstruation. However during menopause, estradiol and progesterone levels in both genders significantly reduced to levels below those in the menstruation phase.

**Table 1:** Female hormone levels throughout the menstrual cycle [16]

Phases	17β-estradiol (nM)	Progesterone (nM)
**Day 1-13 (follicular)**	~0.15-0.37 (40-100 pg/ml)	~0.95 (300 pg/ml)
**Day 14 (Ovulation)**	~0.37-1.47 (100-400 pg/ml)	~0.95-9.54 (300-3000 pg/ml)
**Day 15-28 (luteal)**	~0.15-0.92 (40-250 pg/ml)	~9.54-31.81 (3000-10,000 pg/ml)

#### Delivery of sex hormones to estrogen receptors in the lungs via SHBG

Sex hormone binding globulin (SHBG) is an important steroid hormone binding protein in human plasma and regulates sex hormone delivery to tissues and cells [17]. Plasma SHBG is produced primarily in hepatocytes, which is a glycosylated isoform of SHBG [18] and is produced by the Sertoli cells [19]. In biological fluids, SHBG exists as a homodimer with a separate steroid-binding pocket and a calcium-binding site in each monomer [20] and binds to both androgens and estradiol with nanomolar affinities [21]. In normal men and women, between 40-65% of circulating testosterone (T) and between 20-40% of circulating estradiol (E2) is bound to SHBG [22]. SHBG regulates tissue delivery of sex hormones by binding them and retaining them in the circulatory pool, where they are relatively inert. However, once the sex hormones dissociate from SHBG, they can escape the blood stream, and bind with the intracellular androgen or estrogen receptors, causing changes in gene expression of cells [17]. It is thus generally accepted that only the non-bound hormone is biologically active [23]. SHBG is considered as an estrogen amplifier because T preferentially binds with SHBG, causing more E2 to remain unbound [17].

#### Sex hormone receptors: lung physiology

For many years, we have known that estrogen and progesterone receptors are responsible for sexual development [24] but their effect beyond the reproductive system is becoming increasingly recognized. There are two types of estrogen receptors (ER), two types of progesterone receptors (PR): ER-α, ER-β, PR-A and PR-B, all of which are expressed in rats [25], mouse [26], and humans [27]. While androgen receptor (AR) is expressed primarily in mammalian reproductive tissues [28], ER-α, ER-β, PR-A and PR-B expression have been noted in not only in the mammalian female and male reproductive tracts, but also in the female mammary glands, bone, cardiovascular tissues, lung, and the brain [14,24]. In the lungs, the expression of ER-β protein is twice as that of ER-α [29]. Most of the expression is found in the cytoplasm but minor expression has also been noted in the mitochondria and nucleus [29]. ER-α and ER-β belong to a super-family of nuclear hormone receptors, many of which are ligand-activated transcription factors that regulate gene expression by binding to the promoter region of genes [24]. These receptors contain an N-terminal DNA binding domain and a C-terminal ligand binding domain (LBD) for estrogen [24]. Studies in mice in which ER-α and ER-β were deleted revealed that both types of ER are required for the formation of complete alveolar units in females. ER-α ensures that the lungs differentiate properly during development, leading to normal numbers of alveoli per surface area. ER-β, on the other hand, modulates development of extracellular matrix, leading to normal elastic tissue recoil pressure in the lungs [30,31]. Furthermore, ER-α, but not ER-β, has been demonstrated to mediate an anti-inflammatory response when challenged by carrageenan (CAR) in mice [32]. Upon activation by E2, ER-α inhibited the intracellular trafficking of nuclear factor-κB, thereby preventing the expression of inflammatory genes [33].

#### Mechanism of estrogen receptor activation in the lungs

The exact mechanism by which E2 modulates cell signaling pathways is not completely known and there may be multiple cell signaling pathways by which sex hormones affect gene regulation and expression. According to the free hormone hypothesis, unbound sex steroids freely diffuse across cell surface membranes [23]. Binding of estrogen to the ligand binding domain of the ER causes a conformational change in the receptor, which results in dimerization of ER and translocation to the nucleus [24]. The activated receptor/DNA complex then binds to specific promoter sequences of DNA called hormone response elements (HREs) and recruits other cofactors from the nucleus, which results in transcription of DNA downstream from the HRE [24]. An alternate hypothesis suggests that even E2 bound to SHBG are metabolically active. It is now well recognized that sex steroids target tissues containing membrane-binding sites that can bind with SHBG [34]. In prostate [35] and breast cancer cells, it has been shown that by binding to this site, SHBG triggers cAMP-dependent signaling causing upregulation of adenyl cyclase and other downstream signaling molecules. It is important to note that these data were generated *in vitro *using isolated epithelial cells and not in the context of these cells *in vivo*. Thus, the exact cell signaling pathway of sex hormones remains unknown.

## Discussion

### How sex hormones may modulate COPD

#### Sex hormone on cigarette smoke metabolism

A variety of animal models have been used to examine potential sex-related differences in the risk of inflammatory lung diseases. For example, chronic exposure of mice to cigarette smoke has led to the development of emphysematous-like changes in alveolar structure, and these changes develop more rapidly in females than in males [9]. It is important to note that cigarette smoke is made up of more than 4,000 different chemicals. After inhalation, these chemicals are metabolized in two different phases: Phase I and Phase II. Phase I is mediated largely by cytochrome P450 (CYP) enzymes, which are a family of xenobiotic enzymes that are responsible for de-toxifying cigarette smoke and other environmental irritants into intermediate metabolites. These metabolites in turn are conjugated by Phase II enzymes and excreted. The rate-limiting step in most cases is Phase II. Thus, if there is under-expression of Phase II enzymes or complete saturation of their binding sites, CYP-based metabolites accumulate in the lung. Because some intermediate metabolites are as toxic and others are even more toxic than their parent constituents, lungs may suffer oxidant damage through a process called bioactivation unless there is excellent co-ordination of Phase I and Phase II enzymes. Estradiol up-regulates CYP enzymes without necessarily altering the expression of Phase II enzymes, making the female lungs more susceptible to oxidant damage in response to cigarette smoke. This concept is supported by animal experiments. For instance, Van Winkle et al. showed that the lungs of female mice were more susceptible to naphthalene, a prominent component of side stream cigarette smoke, compared to male mice [36]. Female lungs of rats had higher expression of CYP enzymes and demonstrated increased accumulation of potent oxidants from naphthalene metabolites [37]. Interestingly, in humans, the two CYP enzymes that are up-regulated by cigarette smoke are CYP1A1 and CYP1B1, which are regulated by ER-α [38]. Stimulation of estrogen receptors in the lungs increases protein expression of CYP1A1 by twofold [39]. CYP1A1 has been demonstrated to have high activity for 17β-estradiol 2-hydroxylation, followed by 15α-, 6α-, 4-, and 7α-hydroxylation [40]. 2-hydroxylated estrogens are suggested to be anti-carcinogens, whereas 4- and 16α-hydroxylated estrogens may enhance cancer development [41-43]. Interestingly, 2-hydroxyestrogen but not 16α-hydroxyestrogen has increased clearance rate from the circulation of premenopausal females smokers [44,45], suggesting a differential effect of estrogen metabolism. The increased CYP expression is related to increased levels of estradiol [46] and increased metabolism of cigarette smoke to generate oxidants/oxidizers [47], suggesting that female sex hormone contributes to oxidative stress and greater airway injury.

### How sex hormones may modulate asthma

#### Animal Models for Asthma

When challenged with allergen, female compared to male mice mount an enhanced allergic response, characterized by increased serum levels of ovalbumin-specific IgG1 and IgE [48] and increased airway inflammation [49]. Ovariectomized rats, on the other hand, are protected from increased airway inflammation related to allergens [50]. Estrogen replacement in these ovariectomized rats reestablishes airway inflammation to levels found in intact females [50]. Collectively, these data suggest that female hormones augment airway inflammation in the presence of allergens. One potential mechanism for this response is estrogen-mediated deviation in helper T cells towards a T_H_2 phenotype. Another mechanism is that female hormones down-regulate the expression of regulatory T (T_reg_) cells [51], which play an important role in regulating T_H_2 responses [52]. Estradiol may also up-regulate early phase pro-inflammatory cytokines such as IL-1β and TNF-α and down-regulate anti-inflammatory cytokines such as IL-10 [53]. Consistent with this notion, Huber and Pfaeffle showed that male mice transfected with Coxsackievirus B3 predominantly mounted a T_H_1 cell phenotypic response, while female mice mounted a vigorousT_H_2 cell phenotypic response [54]. Interestingly, when male mice were given estradiol, the ratio of IL-2/IFN-γ to IL-4-producing cells became nearly equal, suggesting that estrogen promotes a T_H_2 response [54]. Progesterone, on the other hand, significantly increases the expression of IL-10, IL1-β, and TNF-α in the lungs, and augments the release of IL-4 by bone marrow cells [53], which may lead to eosinophilia. The existence of such dual hormone effects suggests that the balance between estradiol and progesterone may be critical in host responses to environmental allergens [53]. It should be noted, however that the ovary generates a large number of cytokines and other factors that modulate the inflammatory process and affect the actions of estradiol and progesterone [55,56].

#### Sex hormones and adaptive immunity

Differences in male and female immune responses have been recognized for some time [54]. Females generally mount better humoral immunity than males, while males demonstrate enhanced cellular immune responses [57,58]. These differential immune responses may be important in autoimmune diseases. Helper T cells originate from hematopoietic stem cells in the bone marrow, mature in the thymus and act in many different tissues and organs. The two major subsets of helper T cells are T_H_1 and T_H_2 cells. Naïve T cells differentiate into T_H_1 cells in the presence of IFN-γ and IL-12, which are secreted by dendritic cells in response to bacterial and viral challenge. T_H_1 cells produce a variety of different cytokines including IFN-γ and IL-2 and regulate the cell-mediated immune response [59,60]. In response to extracellular parasites and allergens, dendritic cells produce IL-4, which promotes the T_H_2 lineage. T_H_2 cells, dissimilar to T_H_1 cells, produce mostly IL-4, -5, -10 and -13 to promote IgG, IgA, and IgE antibody isotype responses [61].

Unlike the animal models, immunological modulation by sex hormones in human inflammatory diseases is more complex. However, there are data suggesting that certain inflammatory diseases can modulate the T_H_1/T_H_2 response in humans. For example, patients with systemic lupus erythematosus (SLE) demonstrate a unique pattern of estrogen production and metabolism [62]. SLE is characterized by elevated aromatase enzyme activity and cytochrome p450 isoenzyme (CYP1B1), increased expression of the CD4^+ ^T_H_2 response and a relative under-expression of the CD4^+ ^T_H_1 response [62]. Estrogen is known to be an inducer of aromatase that converts androgen to estrogens. CYP1B1 then converts estrogen to 16α-hydroxyestrone, which is the most biologically active serum estrogen with the most potent immunomodulatory effects [62]. Estrogen can also stimulate secretion of IL-4, -5, -6, and -10 by T_H_2 lymphocytes, while androgens promote the production of IL-2 by T_H_1 cells [62]. Taken together, the relative balance between estrogen and androgen in the circulation may influence the T_H_1/T_H_2 lineage and modulate the overall inflammatory response.

#### Clinical overview of asthma

It is well known that before puberty the incidence of asthma is higher in boys than in girls, but following puberty, the pattern switches such that by adulthood, the prevalence of asthma is nearly 50% higher in women than in men [63]. It is also well known that asthma severity fluctuates over the course of the menstrual cycle [64]. The incidence of asthma tends to decrease after menopause [65] but hormone replacement therapy following menopause is associated with an increased risk of asthma in non-smokers but not with newly diagnosed COPD [66,67], suggesting that sex hormones may play a more important role in the development and progression of asthma.

#### Sex hormones on lung function in asthma

Both forced expiratory volume in one second (FEV_1_) and forced vital capacity (FVC) are the lowest following ovulation in the peri-ovulatory phase of the menstrual cycle. During this phase, circulating estradiol levels are relatively high and progesterone levels are moderate. FEV_1 _and FVC are the greatest during the peri-menstrual period when estradiol and progesterone levels are at their lowest [68]. Consistent with the observation, Zimmerman and colleagues showed that the highest rates of emergency department visits for asthma occurred during the pre-ovulatory phase of the menstrual cycle [69]. Although the exact mechanism for these observations are unknown, as previously stated, both progesterone (at any concentration) and estrogen at high physiological concentrations promote a T_H_2 phenotype, which is likely to be important in asthma pathogenesis.

#### Sex hormones on exhaled nitric oxide in women

An important biomarker of airway inflammation in asthma is nitric oxide (NO). NO is synthesized by activated alveolar macrophages (AM) through the actions of an inducible form of nitric oxide synthase (iNOS) [70]. AM effects bacterial and viral killing by releasing NO and other reactive oxygen and nitrogen species [71]. However, in asthma, this process is dysregulated such that even the absence of microbial products, NO pathways in the airways are enhanced.

Provocatively, in a small study of pre-menopausal women not using oral contraceptives, Mandhane et al. showed that exhaled NO concentrations were positively related to serum progesterone (p < 0.05) but inversely related to serum 17β-estradiol levels [72]. Similarly, Farha et al showed that exhaled NO concentrations were highest in the luteal phase (when serum progesterone levels are expected to reach their peak). Suppression of the menstrual cycle with the use of oral contraceptives, on the other hand, abolished these relationships. Together, these data suggested that progesterone may be a very important regulator of airway inflammation in female asthmatics.

### How sex hormones may modulate Cystic Fibrosis

#### Sex hormone on mucus production

Accumulation of thick, tenacious mucus is a hallmark of cystic fibrosis (CF) and has a central role in CF pathophysiology [73]. Mucus clearance plays an important role in innate immunity in the mammalian lung [74]. Excess sputum production in the lung is one of the key factors in overwhelming ciliary clearance and obstructing the airways, thereby contributing to morbidity and mortality in CF and other inflammatory lung diseases [75]. Mucins are upregulated by pathogens, inflammatory mediators, and toxins, which when dysregulated can exacerbate chronic airway diseases [76,77]. Expression of mucin genes is increased by inflammatory mediators, such as lipopolysaccharide (LPS) [78], TNF-α [79], IL-1 [79], IL-17 [80] and β neutrophil elastase [81]. In addition to these inflammatory mediators, it is known that sex hormones such as estrogen can also up-regulate MUC5B gene expression in normal human airway epithelial cells [82]. MUC5B is one of the major mucins in the human airway submucosal glands [83]. Estrogen is not the only regulator of MUC5B but also regulates a wide diversity of genes involved in extracellular matrix, general cell growth, and differentiation processes [24]. Taken together, estradiol may have the potential to augment mucin production resulting in reduced clearance in CF.

#### Sex hormone on Ca^+2^-activated Cl^- ^secretion

An in-vitro study [84] has shown that high circulating levels of estradiol reduces Ca^+2^-activated Cl^- ^secretion by airway epithelial cells in culture, thereby disrupting ion and water balance and leading to thick, tenacious mucus. Clinical studies have confirmed that Ca^+2^-activated Cl^- ^secretion is decreased in women with CF at times when 17β-estradiol levels are high [69]. Estadiol mediates this effect by inhibiting Ca^+2 ^influx and signaling in both non-CF and CF airway epithelia [74]. Moreover, estrogen also appears to inhibit the uridine triphosphate-mediated Cl^- ^secretion in both women with CF and normal, healthy women [74]. Experiments revealed that a four-fold increase in estradiol was accompanied by a 50% inhibition of UTP-stimulated Cl^- ^secretion in vivo [74]. Since the CF lung disease is characterized by poor Cl^- ^secretion and water retention in the airways [74], it is likely that the estrogen-related disturbance in Cl^- ^secretion via the Ca^+2^-activated channels and other pathways can cause marked perturbations in mucociliary clearance, and accumulation of thick mucus in the airway, resulting in disease progression [85].

#### Sex hormones on lung function in CF

As with asthma, menstrual cycles may also affect lung function in female patients with CF but not in the same phasic manner [86]. Unlike in asthmatics who demonstrate best lung function in the peri-menstrual period, female CF patients have the highest lung function during the luteal phase and the lowest lung function during the pre-ovulatory phase [87]. Johannesson showed that FEV_1 _was 66% of predicted in female CF patients during the luteal phase, while it was only 63% during ovulation (P < 0.01) [86], which interestingly is associated with increasing levels of estradiol. The differences in the swings in lung function during the menstrual period between asthmatics and CF patients may relate to the relative importance of mucus production in their pathophysiology. Although mucus production and clearance are thought to be important both diseases, in CF, it is has a pre-eminent role, while in asthma, it is believed to have a more complementary role in disease progression. By up-regulating MUC5B expression, high estradiol levels during the pre-ovulatory phase may significantly enhance mucus production (through the mechanisms discussed in the previous section) and lead to worsening of airflow limitation in CF. Furthermore, 17β-estradiol in high physiological concentrations inhibits local production of IL-8 by up-regulating secretory leucoprotease inhibitors [88], which may diminish the ability of the female respiratory tract to contain bacterial infections in CF.

## Summary

Women are relatively more prevalent in the epidemiology of asthma, CF and COPD and appear in general to have worse prognosis than their male counterparts. The exact mechanism of this process is still uncertain. Emerging data suggest that female sex hormones play a role in these inflammatory airway conditions, through different but related mechanisms. Studies have shown that estrogen promotes a T_H_2 response, while androgen promotes a T_H_1 response, which may be relevant in asthma. Estradiol inhibits Cl^- ^secretion in the CF lung and up-regulates mucus production, which may be very relevant in CF. Cigarette smoke is de-toxified through Phase I and Phase II enzymes and estrogens may preferentially up-regulate Phase I enzymes, leading to accumulation of toxic metabolites through a process called bioactivation. This may be relevant in the pathophysiology of COPD. Although less well studied than estrogen, progesterone may also play relevant roles in inflammatory airway disease by amplifying airway inflammation. With the rise in the burden of these diseases in women worldwide, there is a pressing need to better understand the biological roles of sex hormones in modulating airway inflammation, mucus production and cigarette de-toxification and other processes relevant to COPD, asthma and CF.

## Abbreviation List

CF: cystic fibrosis; COPD: chronic obstructive pulmonary disease; E2: estradiol; ER: estrogen receptor; HRE: hormone response element; IL: interleukin; MUC5B: mucin 5B; NO: nitric oxide; PR: progesterone receptor; SHBG: sex hormone binding globulin; TNF: tumor necrosis factor

## Competing interests

Anthony Tam - declared no competing interests

Don Morrish - received funding from GSK/CIHR, and Wyeth Pharmaceuticals.

Samuel Wadsworth - declared no competing interests

Delbert Dorscheid - declared no competing interests

Paul S F Man - received educational grants from Glaxo-Smith-Kline and Astro-Zenecca to support research

Don D Sin - holds a Canada Research Chair in COPD and a senior clinical scholarship with the Michael Smith Foundation for Health Research (MSFHR) and has received grants from Wyeth Pharmaceuticals, GlaxoSmithKline, AstraZeneca and Pfizer.

## Authors' contributions

AT participated in drafting the manuscript. DM, SW, DD, SFPM, and DDS participated in critical revisions. All authors read and approved the final manuscript.

## Pre-publication history

The pre-publication history for this paper can be accessed here:

http://www.biomedcentral.com/1472-6874/11/24/prepub
